# Knowledge and barriers to implementing dementia risk reduction among older people and health professionals in community daycare centers

**DOI:** 10.1002/alz.092537

**Published:** 2025-01-09

**Authors:** Jose M Aravena, Waldo Torres, Javiera Gallardo, Fernanda Sobarzo, Gillian Saavedra, Rocio Briceño, Camilo Miranda, Becca R Levy

**Affiliations:** ^1^ Department of Social & Behavioral Sciences, School of Public Health, Yale University, New Haven, CT USA; ^2^ Yale University, New Haven, CT USA; ^3^ Universidad Central de Chile, Santiago Chile; ^4^ Universidad de Chile, Santiago Chile; ^5^ Servicio Nacional del Adulto Mayor SENAMA, Santiago Chile

## Abstract

**Background:**

Assessing knowledge of dementia prevention from both the individual (patients) and the structural level (health professionals) in the same settings is vital to implementing dementia risk reduction programs. However, most studies have only focused on one level. Thus, this was the aim of our study.

**Method:**

A qualitative study was conducted with two populations: patients aged 60 and older with no dementia and health professionals, attending and working at community daycare centers for older adults in Chile, respectively. People from 10 different daycare centers were invited to participate in two separate focus groups per center (patients: 5‐15 participants; health professionals: 3‐7 participants). Focus groups included the topics of cognitive changes of aging and dementia, dementia risk factors, and barriers to brain health. Thematic analysis following grounded theory and inductive analysis was performed to generate main themes.

**Result:**

In 19 focus groups, 115 patients and 51 health professionals participated. In the main results, older adults frequently mentioned memory problems as a *‘normal part of aging’* and Alzheimer’s disease as *‘the severe stage of dementia.’* Health professionals recognize signs and symptoms of dementia. Both groups agreed that uncertainty about memory loss and Alzheimer’s disease can generate anxiety and isolation in older people. Patients showed more agreement on dementia risk factors than health professionals and were able to mention risk factors not mentioned by health professionals (e.g., TBI, air quality, neighborhood factors). Patients from high‐income municipalities were more prone to attribute Alzheimer’s disease to genetic factors than patients from other municipalities, but health professionals showed no differences. Interestingly, patients used popular figures in media with a diagnosis of dementia (e.g., Ronald Reagan) as examples of why *‘Alzheimer’s disease can’t be prevented.’* Structural barriers to brain health were similar between groups (negative experiences with healthcare and ageism). Patients referred to intergenerational contact and health professionals increasing awareness among patients' families as opportunities for brain health.

**Conclusion:**

Health professionals highlighted the need for updates and homogenization regarding dementia risk reduction. Patients’ knowledge may be influenced by social determinants and portraits of aging and brain health. Addressing structural factors could benefit dementia prevention in both groups.

